# Another Criticism of Dr. Preston’s Case

**Published:** 1890-09

**Authors:** Clarence N. Payne

**Affiliations:** Bridgeport, Ct.


### ANOTHER CRITICISM OF DR. PRESTON'S CASE.

EDITORS HOMŒOPATHIC PHYSICIAN:

I desire to criticise the diagnosis of the case reported by Dr. Mahlon Preston, as a case of syphilis, in the August, 1890, number of THE HOMŒOPATHIC PHYSICIAN.

I think it will be evident to every well-informed man, from the Doctor's own description of his case, that it was one of simple local non-infective venereal sore or *chancroid*, and that there is no evidence of anything of a true syphilitic nature about it.

This is very plain from two prominent facts which he mentions, if from nothing else about the case.

*First.* He says, "My earliest knowledge of the present case dates to within ten days of first exposure." Now we know that the primary lesion of syphilis does not make its appearance till the third to fifth week after exposure, while that of a soft chancre or venereal ulcer may appear in forty-eight hours.

*Second.* He says there were "three large and deep chancres. * * * These ulcers were eroding rapidly on the edges," etc. This is surely not the picture of the primary lesion of syphilis which we know to be a *single* indurated chancre.

From the above I think it is but fair to answer his query, "Have I a right justly to claim that a radical cure has been made," in the negative, so far as its being a case of syphilis which was cured is concerned. Respectfully,

CLARENCE N. PAYNE.

BRIDGEPORT, CT., August 7th, 1890.

